# Conflict with Parents in Adolescent Depression: Associations with Parental Interpersonal Problems and Depressive Symptoms

**DOI:** 10.1007/s10578-020-00955-0

**Published:** 2020-01-18

**Authors:** Erling W. Rognli, Luxsiya Waraan, Nikolai O. Czajkowski, Ole André Solbakken, Marianne Aalberg

**Affiliations:** 1 Department of Psychology, University of Oslo, Postboks 1094 Blindern, 0317 Oslo, Norway; 2 Division of Mental Health Services, Akershus University Hospital, Lørenskog, Norway; 3 PROMENTA Research Center, Department of Psychology, University of Oslo, Oslo, Norway; 4 Division of Mental Health, Norwegian Institute of Public Health, Oslo, Norway

**Keywords:** Parent-adolescent conflict, Adolescent depression, Parental depression, Interpersonal circumplex, Bayesian data analysis

## Abstract

Conflict with parents is common among depressed adolescents, interferes with treatment, and may increase risk of recurrence. Parental depressive symptoms have been shown to predict conflict with adolescent children, but an important role for different kinds of parental interpersonal problems, as described by interpersonal circumplex, is also plausible. This study compared parental interpersonal problems to parental depressive symptoms as predictors of parent-adolescent conflict reported by a depressed adolescent child, using multilevel linear regression, leave-one-out cross-validation and model stacking (N = 100 parents, 57 mothers and 43 fathers, of 60 different adolescents). Cross-validation and model stacking showed that including parental interpersonal problems contributes to accurate predictions. Parents reporting more interpersonal problems related to excessive dominance or submissiveness was associated with increased or decreased conflict, respectively. Parental depressive symptoms were found to be negatively associated with parent-adolescent conflict only in father-adolescent relationships.

**Electronic supplementary material:**

The online version of this article (doi:10.1007/s10578-020-00955-0) contains supplementary material, which is available to authorized users.

## Introduction

Parent-adolescent conflict is common between depressed adolescents and parents of both genders [1, 2]. High levels of parent-adolescent conflict predicts the development of adolescent depression [3–5], appears to interfere with treatment [6, 7], and increases the risk of recurrence in adulthood [8]. Both maternal and paternal depression are well established as predictors of parent-adolescent conflict [9–11], but as managing and resolving conflict is inevitably an interpersonal situation, an association with parental difficulties in interpersonal functioning is also plausible.

### Interpersonal Theory and the Interpersonal Circumplex

A prominent approach to individual differences in interpersonal functioning is interpersonal theory, originating in the work of Sullivan [12]. This line of research has identified two fundamental dimensions of interpersonal phenomena, termed agency and communion. These two dimensions and the interpersonal circumplex they define when combined has shown good fit to variation in observed interpersonal behaviour, as well as interpersonal styles and individual differences in interpersonal functioning [13, 14]. As an interpersonal disposition, agency concerns being predominantly dominant or submissive across interpersonal situations, while the dimension of communion in a similar manner refers to being predominantly nurturing and warm or more distant and cold. The interpersonal circumplex has the advantage of not assuming interpersonal difficulties to be unidimensional, allowing for the impact on functioning to differ across kinds of interpersonal situations.

### Interpersonal Problems and Parent-Adolescent Conflict

In adolescence, the development of age-appropriate autonomy requires gradual renegotiation of patterns of interaction, and parent-adolescent conflicts are suggested to play an important role in this reorganization of the parent–child relationship [15, 16]. In interpersonal theory terms, adolescents will tend to assume high-agency interpersonal behaviours across an increasing range of interpersonal situations with their parents, both conflictual and non-conflictual. Interpersonal theory predicts that if parents reciprocate with low-agency behaviours to an appropriate degree, the result is a transitory increase in interpersonal conflict, before a new pattern of interaction is established [14]. Such patterns of transitory increase in conflict and subsequent realignment of the relationship has been observed in non-clinical samples [16]. The functional impairment and cognitive and affective symptoms of adolescent depression will tend to increase the frequency of potential conflicts in the parent-adolescent relationship, as adolescents are unable to meet parental expectations and behave in ways parents might find unacceptable [17]. Parental difficulties in interpersonal functioning could then lead to a cascade of parent-adolescent conflict, first making parents more prone to escalate potential conflicts, and then increasing the probability of negative resolution and persistence of these conflicts.

### The Present Study

Interpersonal theory is a conceptually rich and well-developed theoretical framework for studying parent-adolescent interaction [18], but has not yet been applied to the study of parent-adolescent conflict in adolescent depression. The aim of the present study is to evaluate whether parental interpersonal problems are associated with parent-adolescent conflict reported by their depressed adolescent children and investigate whether the strength of the association varies across the interpersonal circumplex. We will also assess whether any such association has incremental predictive value compared with the expected association between parental depression and parent-adolescent conflict that has been found in previous research in related populations [10, 19–21].

## Methods

### Participants

The data analysed in this study are from baseline assessments in a randomized controlled trial (clinicaltrials.gov identifier NCT01830088) comparing Attachment-Based Family Therapy [22] to treatment as usual [23], manuscript in preparation]. Participating families were recruited among adolescents referred to two Child and Adolescent Mental Health Services (CAMHS) in South-eastern Norway. During pre-specified recruitment periods, all referral letters for adolescents (13–17 years) were examined for mentions of depression or core depressive symptoms (depressed mood, anhedonia, or fatigue). The CAMHS routinely administered the Youth Self Report [24], and these were screened for raw scores on the Affective Problems subscale above six to find depressed adolescents not identified as such in their referral letters [25]. Eligible adolescents or their parents, depending on adolescent age, were contacted by telephone and invited to participate in a randomized trial of family therapy for adolescent depression. 276 adolescents were contacted. Participants were required to be currently living with an adult who had become a caregiver for them before age four, and willing to have this adult participate in treatment. Interested adolescents meeting these criteria (160 of 276) were screened with Beck Depression Inventory-II [26] over telephone and invited for an assessment session if they scored above 17, a threshold expected to maximize sensitivity [27]. Of those screened with the BDI-II, 136 scored above the threshold, and 100 of these agreed to meet with study personnel for a clinical assessment. Adolescents were included in the study if they scored above 15 on the Grid Hamilton Depression Rating scale [28] and met Diagnostic and Statistical Manual of Mental Disorders [29] criteria for a current major depressive episode. Adolescents meeting criteria for a psychotic disorder, eating disorder, bipolar disorder, intellectual disability or pervasive developmental disorders were excluded from the study. One family withdrew consent after assessments had been completed. In all 60 adolescents were included (55 female, 5 male), with 43 fathers and 57 mothers, among whom there were 19 intact couples.

### Procedures

Participating adolescents and their parents met with a study-affiliated clinical psychologist (the first or second author) at the CAMHS for an assessment and written informed parental consent and adolescent assent was obtained. Adolescents and parents were then interviewed separately. All interviews were video recorded. Self-report measures collected from parents and adolescents were completed during the appointment.

### Measures

#### Parent-Adolescent Conflict

Parent-adolescent conflict was measured by the report of the adolescent on the Perception of the Dyad subscale of the Conflict Behavior Questionnaire [CBQ, 30], separately for each parent. This scale consists of 16 items rated true or false concerning current conflict in a parent-adolescent relationship. The CBQ has been widely used as a measure of parent-adolescent conflict in depressed adolescents [e. g. 31, 32]. The CBQ was translated to Norwegian for this study, and a blind reverse translation was approved by the original author.

#### Parental Interpersonal Problems

Parental interpersonal problems were measured by parents completing the Inventory of Interpersonal Problems—Circumplex [IIP-C, 32 item version, 33, 34]. The items of IIP-C map onto the interpersonal circumplex, and is well established as a valid and structurally sound measure of problems in interpersonal functioning [35, 36]. We computed scores for each parent on the two main orthogonal factors Agency and Communion, and the general interpersonal distress factor Elevation, according to the method described by Gurtman and Balakrishnan [37], using available Norwegian norms for standardizing the scores [38]. An unofficial Norwegian translation of the IIP-C was used, with some items deviating slightly from the official Norwegian translation. We carefully examined item-scale correlations and found that the circumplex structure of the instrument was not compromised.

As a norm-adjusted standardized variable, Agency runs from negative scores for more problems than the mean of the normative sample related to being interpersonally submissive, through zero for the mean level of interpersonal difficulty, to positive scores for more problems related to being interpersonally dominant. Similarly, Communion runs from negative scores for problems related to being withdrawn and cold, to positive scores for more problems related to being preoccupied with caring and maintaining interpersonal closeness. These main factors of the IIP-C are stable measures of a trait-like interpersonal style [39]. Elevation is a measure of a more state-like general level of interpersonal distress [37].

#### Parental Depressive Symptoms

Parental depressive symptoms were measured by parental responses to 17 items from the Symptom Checklist 90—Revised [SCL-90-R, 40], which comprise the revised depression subscale developed by Paap and colleagues [41] using nonparametric item response modelling and a large Norwegian outpatient sample.

#### Adolescent Depression Severity and Diagnosis

Diagnostic assessments were conducted with the Schedule for Affective Disorders and Schizophrenia for School-Age Children-Present and Lifetime Version [42]. The severity of adolescent depressive symptoms were further assessed with the clinician-rated Grid-Hamilton Depression Rating Scale [28].

### Analysis Plan

We conducted analysis within a Bayesian modeling framework, with estimation by Hamiltonian Monte Carlo (HMC) as implemented in the Stan programming language, using the RStan package version 2.18.2 [43], for R version 3.5.1 [44]. The results of a Bayesian analysis are distributions that show the probability of different model parameter values, conditional on the data and the model. For the reader unfamiliar with Bayesian statistics, Baldwin and Larson [45] provide a very accessible introduction to the use of Bayesian linear regression in clinical psychology. Bayesian modelling is also well suited to small sample sizes, as long as proper caution is paid to choice of priors and validation of convergence [46]. Stan and R code for the analysis, as well as the sets of samples drawn from the posterior distribution and used for inference, has been made available at https://doi.org/10.17605/OSF.IO/D2F8A.

#### Modelling Predictors of Parent-Adolescent Conflict

Our overall analytic approach was multiple regression modelling, with adolescent report of parent-adolescent conflict as the dependent variable, and a simple multilevel structure with parents nested within adolescents and a random intercept for each adolescent [47]. The regression models were specified with a latent dependent variable, obtained by fitting a two-parameter logistic item response model to the responses on the CBQ Perception of the Dyad scale. Stan is well suited for estimating item response theory (IRT) models, and these can be incorporated as part of a larger model of interest [48]. Our aim in doing IRT modelling was not to develop a revised measure, only to extract a continuous and more reliable dependent variable. Another advantage of item response models is that the reliability of the scale can be evaluated across the range of the latent trait, showing at what ranges the scale provides most information, and hence highest precision, given an item response models that fits the data [49].

Given that parent-adolescent conflict is clearly a multi-determined phenomenon, we expected observations that deviated substantially from the predicted value based on a limited set of predictors. We therefore aimed for robust estimation of the regression model, by defining a t-distribution for the likelihood, with the degrees of freedom estimated as a parameter [50]. This allows the model to adapt the level of robustness to the data, and hence avoid letting such outlier observations influence the slope too much.

Bayesian analysis requires specification of a prior distribution for all parameters (priors), representing our assumptions and knowledge about the model parameters independently of the data. For example, if a standardized beta coefficient from a linear regression model could not reasonably be expected to be greater than 2 or smaller than − 2, and would most likely fall between − 1 and 1, as would often be the case in clinical psychology, this knowledge can be encoded by a Normal (0,1) prior distribution. A reader evaluating the results of a Bayesian analysis should consider the priors specified and decide whether they are reasonable, and priors should hence always be reported [45]. The priors for this analysis are summarized in Table 1.

**Table 1:** Prior distributions and reasoning for choices of prior

Parameter	Prior distribution	Reasoning
Random intercepts	Hierarchical normal prior, with location 0 and a Half-student’s t (3, 0, 1) hyperprior for scale	Defines random intercepts as deviations from the sample mean of 0, and estimates the variance of the random intercepts from the data, with a weakly informative hyperprior
IRT-theta (Conflict level)	Normal (0,1)	Fixes the location and scale of the latent conflict variable for model identifiability, and to ensure a standardized dependent variable for interpretability
IRT-beta (Item difficulty)	Hierarchical normal prior with hyperpriors Normal (0, 3) for location and Half-student’s t (3, 0, 1) for scale	Weakly informative hierarchical prior, as the interdependent IRT-theta parameter has fixed location and scale
IRT-alpha (Item discrimination)	Gamma (2, 0.5)	Item discrimination parameters for the CBQ assumed to lie between 0 and 10, as the item characteristic curve does not change meaningfully across alphas larger than 10
Error variance in regression model	Half-student’s t (3, 0, 1)	Regularizing prior on the error variance, which still allows for large estimates if warranted by the data
Degrees of freedom in Student’s t-distributed likelihood	Gamma (2, 0.1) Constrained to be ≥ 1	Degrees of freedom for the likelihood between 1 and about 30, allowing for the likelihood to be very near normal, or have a large degree of robustness, as required
Regression coefficients	Normal (0, 1)	Regularizing prior on the regression coefficients

#### Missing Data Management

The CBQ had 0.7% data missing as single items. For the cases with items missing on the CBQ, we estimated the latent variable based on the observed items. There was 0.4% data missing as single items from the IIP-C, and 0.1% from the SCL-90 Depression Scale. For the individual IIP-C scales and the SCL-90 Depression scale we singly imputed missing responses to items by two-way imputation [51], using the ‘twoway’ function from the R-package ‘mokken’ version 2.8.11 [52], before calculating scale scores. To verify that single imputation was appropriate, we multiply imputed 1000 datasets using two-way imputation and calculated the variables Agency, Communion, Elevation and Parental depressive symptoms in each dataset for all respondents with missing responses. This allowed us to assess to what extent the calculated summary variables of interest to us varied across imputations. The standard deviations of the standardized summary variables calculated across imputations and within each respondent ranged from 0.02 to 0.04 for Agency, 0.02 to 0.04 for Communion, 0.01 to 0.02 for Elevation and < 0.01 to 0.04 for Parental depressive symptoms, indicating that imputations varied minimally, and that single imputation was unlikely to bias results severely.

In two cases, the complete CBQ was missing, in three other cases the complete SCL-90-R, and in one of these three cases the IIP-C was also missing. For these we used Bayesian imputation, treating the missing observations as unknown parameters of the model, which preserves the uncertainty due to not having made these observations in the posterior distribution [53]. For the missing observations of IIP-C and SCL-90-R, we specified a multivariate normal distribution for the complete predictor matrix, composed of observed data and parameters for the missing observations. This allows us to use any information available in the other predictor variables to inform the estimates for the missing observations.

#### Estimation and Evaluation of Convergence

All posterior samples used for inference were drawn using four markov chains in Stan with the NUTS algorithm, 1000 warmup iterations, and 3500 samples from each chain. There were no divergent iterations or other Stan indicators of biased inference. Gelman-Rubin statistics [50], and effective sample size estimates (see Table 4), indicated convergence for all parameters.

#### Evaluating Hypotheses Through Cross-validation and Model Stacking

Our research question can be framed as a question of comparative predictive value of different models. Does parent report of interpersonal problems contribute unique information to predicting adolescent report of parent-adolescent conflict, when compared with a model predicting conflict from parental report of depressive symptoms? The predictive precision of models can be compared by estimating their expected fit to new data. We estimated this using exact leave-one-out cross-validation. This is conducted by refitting the model once for each observation (or cluster of observations in hierarchical models, if predictive precision for new clusters is what is of interest) with one observation left out for each refitting. The log-likelihood of the held-out data given the refitted model is saved for each refitting, and together estimates the expected log predictive density, a measure of the expected fit of the model to new data from the same distribution [54]. Different models can then be compared on their expected log predictive density values.

The results of leave-one-out cross-validation can also be used for model stacking, a procedure that takes a set of models and gives the weighted combination of these that has the highest expected predictive accuracy [55]. The obtained stacking weights are interpretable as the contribution of each model to predictive accuracy when combined with the other models entered in the stacking procedure.

We fitted, cross-validated and stacked four models. As our model had a hierarchical structure with parents nested within adolescents, we left one family out at a time. To calculate the pointwise log-likelihood, we took the summed log-probability mass of the observed item responses to the CBQ conditional on the expected value from the regression and the item parameter estimates. The first model had parental depressive symptoms as the predictor. The second had parental depressive symptoms, parent gender and their interaction as predictors. The third had the three parent interpersonal problem variables agency, communion and elevation as predictors. The fourth had the three parent interpersonal problem variables, parent gender, and interaction terms between each interpersonal problem variable and parent gender. We also included adolescent age in years, centred on age 15, as a covariate in all four models, as age has been shown to be associated with parent-adolescent conflict [56].

By cross-validating and stacking these models, we can obtain an estimate of the relative predictive value of parental interpersonal problems and parental depressive symptoms for predicting parent-adolescent conflict for a new depressed adolescent, and assess whether any associations are conditional on parent gender, by comparing the fit of models with interaction terms to models without. Due to the low number of male adolescents in the sample, we did not fit models with adolescent gender.

## Results

### Distribution of Predictor Variables

The mean scores on the IIP-C variables were: agency − 0.31 (SD 0.63, range − 2.25; 1.36), communion 0.23 (SD 0.54, range − 1.89; 1.88) and elevation 0.22 (SD 0.69, range − 1.37; 1.66), showing a considerable variation in the degree and kind of interpersonal problems reported by the parents in this sample. On the SCL-90-R revised depression scale (items rated 1–5), the mean item score was 1.94 (SD 0.78, range 1; 4). Some parents reported clearly clinical levels of depressive symptoms: 39 (40.2%) were at or above the mean raw score of a clinical outpatient sample [41]. Posterior estimates of the predictor variable correlation matrix are displayed in Table 2.

**Table 2:** Estimated predictor correlation coefficients (posterior means and 93% CI)

	Parental depression	Agency	Communion
Agency	− .42 (− .55; − .27)		
Communion	− .06 (− .26; .15)	− .22 (− .45; .05)	
Elevation	.58 (.45; .68)	− .54 (− .67; − .36)	− 0.18 (− .43; .12)

### Item Response Modelling of the CBQ—Perception of the Dyad

**Fig. 1:**
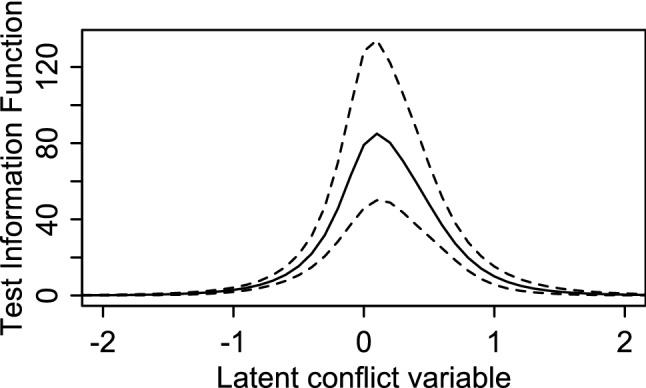
Test information function for the conflict behaviour. Questionnaire—perception of the Dyad

Inspection of item characteristic curves and the observed data indicated adequate fit. These plots can be found in the Supplementary material. Figure 1 shows the test information function, which indicates that the scale has most information about above-average levels of conflict, but covers the relevant range reasonably well.

### Evaluating Models by Leave-one-out Cross-validation and Model Stacking

The four models and the differences in expected log posterior density are displayed in Table 3, along with the stacking weights obtained from the stacking_weights() function from the R package loo [57].

**Table 3:** Results of leave-one-out crossvalidation and model stacking

Model	Difference	SE	Stacking weight
Parental interpersonal problems	0	0	0.75
Parental interpersonal problems with parent gender interaction	3.89	1.58	0
Parental depressive symptoms	8.35	6.21	0
Parental depressive symptoms with parent gender interaction	10.07	7.28	0.25

Difference = Difference in expected log posterior density to model with highest expected log posterior density; SE = Standard error of the difference; Stacking Weight = Model weight in stacking procedure

Observing the expected log posterior densities and their standard errors, several conclusions may be drawn. Firstly, the data do not support modelling an interaction between parent gender and interpersonal problems, given the difference in expected log posterior density and the stacking weights. Secondly, the data supports both parent interpersonal problems and parental depressive symptoms as predictors of parent-adolescent conflict. Though the difference between the model with parental interpersonal problems and the models with parental depression is larger than the standard deviation, it is not by much. The stacking weights imply that a combination of the model with parental interpersonal problems and the model with parental depression and an interaction with parent gender gives the highest expected predictive accuracy, but with most weight given to parental interpersonal problems.

### Regression Parameter Estimates

The regression model parameter estimates from the two models given a positive stacking weight are displayed in Table 4. Both models also have a large number of hierarchical parameters (such as IRT item parameters and hyperparameters, latent trait estimates, and random intercepts per adolescent). These parameters are summarised in the Supplementary material.

**Table 4:** Regression model parameter estimates

Parameters	Mean	SD	93% CI	ESS	Ȓ
Interpersonal problems model					
Agency	0.19	0.07	0.07; 0.31	3768	1
Communion	0.02	0.06	− 0.08; 0.12	5126	1
Elevation	0.08	0.07	− 0.04; 0.21	6509	1
Adolescent age	− 0.03	0.03	− 0.09; 0.04	7081	1
Variance of errors	0.27	0.06	0.18; 0.39	1016	1
Variance of random effects	0.21	0.07	0.05; 0.34	1214	1
Degrees of freedom in t-likelihood	21.88	14.15	4.84; 53.70	13,611	1
Parental depressive symptoms model					
Intercept	− 0.03	0.12	− 0.24; 0.18	840	1
Depressive symptoms	− 0.16	0.07	− 0.30; − 0.04	4719	1
Depressive symptoms × mother	0.16	0.09	0; 0.33	5495	1
Mother	0.02	0.08	− 0.12; 0.17	9331	1
Adolescent age	− 0.03	0.03	− 0.10; 0.03	8449	1
Variance of errors	0.30	0.06	0.20; 0.41	1639	1
Variance of random effects	0.16	0.08	0.02; 0.30	1610	1
Degrees of freedom in t-likelihood	20.8	13.85	4.57; 51.59	14,430	1

Mean = Posterior mean; SD = Posterior standard deviation; 93% CI = 3.5th and 96.5th percentiles of the posterior distribution; ESS = Effective Sample Size, refers to the effective number of samples from the posterior distribution; R̂ = Gelman-Rubin Statistic, indicates convergence of HMC chains at 1

The regression parameter estimates show a positive association between parental agency-related interpersonal problems and parent-adolescent conflict. The positive sign of the coefficient implies that as parents report more problems related to being too interpersonally domineering, their adolescents will tend to report more conflict. The posterior distribution of regression coefficient values (summarised in the table by its mean, standard deviation and the 3.5th and 96.5th percentiles) shows that the data are not at all consistent with a negative association under this model. The data are also not very consistent with a near-zero association, with only a 0.08 probability of a standardised regression coefficient smaller than 0.1.

The posterior distribution for Communion is symmetric around 0, which means the data are most consistent with no strong association between parent-adolescent conflict and parents reporting difficulties either being too cold and distant or overly concerned with maintaining relationships. It is worth noting that in a Bayesian data analysis, an estimate of 0 is no less certain than any other estimate, unlike in classical hypothesis testing, where failure to reject the null hypothesis cannot be interpreted as evidence for the null hypothesis being true [58].

The posterior mean estimate for Elevation, the interpersonal problem variable measuring general interpersonal distress, is weakly positive, but there is considerable uncertainty in this estimate. An association near zero (between − 0.1 and 0.1) is quite consistent with the data, with a probability of 0.60, but any association is probably positive, with a 0.89 probability of a regression coefficient larger than 0. This means there may be an association between parental general interpersonal distress and parent-adolescent conflict, and that any association is probably positive and of small magnitude, but that the data does not provide conclusive evidence.

For parental depression, the coefficients show a negative association for fathers only, as the positive coefficient for the interaction with dummy-coded parent gender is of similar magnitude as the coefficient for parental depression. The coefficient for paternal depressive symptoms is below − 0.1 with a 0.81 probability. The posterior distribution of the total coefficient for maternal depressive symptoms (obtained by elementwise addition of the posterior samples for the two coefficients) shows evidence for no strong association between maternal depressive symptoms and parent-adolescent conflict, with a 0.92 probability of a coefficient between − 0.1 and 0.1. Both the regression coefficient for parent gender and the intercept (necessary in a model with a dummy-code, to estimate the effect of belonging to the reference category, in this case a father-adolescent relationship), is estimated very close to 0, implying that there are probably no large differences in reported conflict level between mother-adolescent dyads and father-adolescent dyads as groups. The coefficient for adolescent age is also very close to 0 in both models.

In summary, there are two main findings: Adolescent reported conflict is predicted to be higher when parents report more problems than average related to being too interpersonally domineering, and lower when parents report more problems than average being interpersonally submissive, and this applies regardless of parent gender. Given the model weighting, this interpersonal tendency has higher predictive utility than parental depressive symptoms. Conflict is also predicted to be higher with fathers who report less than average depressive symptoms, and lower when fathers report more depressive symptoms, while the depressive symptoms of mothers do not appear to be strongly associated with parent-adolescent conflict.

## Discussion

The aim of our analysis was to evaluate to what extent the interpersonal problems reported by parents are associated with parent-adolescent conflict reported by their depressed adolescent children, and whether these associations varied across the interpersonal circumplex. We also wanted to assess whether any such associations have incremental predictive value compared with the expected association between parental depression and parent-adolescent conflict that has been found in previous research in related populations [10, 19–21]. Our results indicate that parental agency-related interpersonal problems are associated with parent-adolescent conflict, and that parent interpersonal problems does add predictive value.

### Parent-Adolescent Conflict is Associated with Parental Agency-Related Problems

Our results suggest an association between interpersonal problems on the agency-dimension and parent-adolescent conflict. In childhood and early adolescence, resolution of parent-adolescent conflict is mainly by parental power assertion, or reciprocal withdrawal [59]. It has been suggested that conflicts and renegotiation of interaction patterns for conflict resolution is an important mechanism of change in parent-adolescent relationships [15]. Finding parental problems with being too dominant and assertive to be related to increased parent-adolescent conflict is consistent with this view. The Agency variable of the IIP-C indexes difficulties in assuming an interpersonally submissive or dominant position when needed [37]. Parents scoring high on the Agency variable would be expected to struggle with accepting and encouraging age-appropriate adolescent autonomy, and to find the normative transition to increasing interpersonal equality in parent–child conflicts [60], to be particularly challenging. It is worth noting that parents scoring in the negative range on the Agency variable are also reporting above average levels of interpersonal problems, but their problems concern being too submissive and unassertive. These are predicted to have lower than average levels of parent-adolescent conflict, and the model appears to fit equally well across the range of the Agency variable. This means that parental report of more severe difficulties with an unassertive interpersonal style is associated with lower levels of parent-adolescent conflict. While this is not theoretically surprising in itself it demonstrates an important point: If interpersonal difficulties are not differentiated in measurement and modelling, it may obscure specific associations between different interpersonal processes and different dimensions of interpersonal difficulties, such as those described by the interpersonal circumplex. Though they are found to have less conflicts with their depressed adolescent, it is entirely possible that these parents find other aspects of the parent-adolescent relationship, such as limit-setting, more difficult than parents reporting less such problems.

It is also notable that problems relating to preoccupation with closeness and care, or with being withdrawn and detached, do not appear to be strongly related to the level of parent-adolescent conflict. This suggests that the way in which parents respond to the developing autonomy of the adolescent may be more important for the level of parent-adolescent conflict than how they handle closeness and warmth in the parent-adolescent relationship. Still, parental interpersonal problems on the communion dimension may very well be associated with other difficulties in the parent-adolescent relationship that were not assessed in this study.

### Paternal but not Maternal Depressive Symptoms are Associated with Less Conflict

Not finding parental depressive symptoms to be positively associated with parent-adolescent conflict was surprising, given the literature supporting this association, for both parent genders [e. g. 20, 61]. However, there are other discrepant findings in the literature, such as a longitudinal study of an at-risk sample which did not find parental depression to predict conflict trajectory membership [62], and a longitudinal study of mother-adolescent conflict interactions where maternal internalising symptoms was not associated with maternal conflict behaviour [63]. Any explanation for this unexpected finding will nevertheless be speculative. It might be due to differences in measurement and operationalisation of parent-adolescent conflict. In a meta-analysis of the association between paternal depression, father–child conflict and child psychopathology, larger effect sizes were found to be associated with community samples and parent-reported measures of parenting behaviours [20]. A second possibility is discontinuity of the association across populations and contexts, with the dynamics of parental depression and parent-adolescent relationships changing when adolescents themselves develop a depressive disorder. Lastly, although a positive association for both parent genders is quite improbable given these data and the model, improbable is still not impossible, and the sample may simply be unrepresentative.

### Strengths, Limitations and Recommendations for Future Research

This study has several limitations. The sample size is small, but this was somewhat mitigated by making the individual parent the unit of analysis in a multilevel model, and then fitting and comparing models where all predictors interacted with parent gender. As the number of male adolescents in the sample is minimal, replication is necessary to generalise the findings to depressed adolescent males. Further, the study design is cross-sectional. A longitudinal design would have allowed for stronger inferences concerning the direction of effects. However, as the agency and communion factors of the IIP-C has considerable temporal stability [64], and concerns how the respondent perceives their interpersonal functioning across relationships, a strong influence on this measure by current conflict with their depressed adolescent is less plausible.

The study is strengthened by clinical assessment of a major depression diagnosis, by not relying on a single informant, having a large proportion of participating fathers and employing powerful and modern modelling and estimation techniques.

These findings add to the literature by demonstrating how parental interpersonal dispositions are related to parent-adolescent conflict in adolescent depression. They demonstrate the utility of interpersonal theory and the IIP-family of measures for studies of conflict processes in adolescent depression. While not carrying the weight of evidence necessary for any clinical recommendation, we would suggest future studies on conflict processes in adolescence consider including an IIP measure such as the brief IIP-C-IRT [65] as a theoretically rich and differentiated measure of parent and adolescent interpersonal styles.

## Summary

Parent-adolescent conflict is common among depressed adolescents and their parents. High levels of parent-adolescent conflict can interfere with treatment and may increase risk of recurrence. Parental depressive symptoms have been shown to predict conflict with adolescent children, but as management of conflicts is inevitably an interpersonal situation, parental difficulties in interpersonal functioning could also play an important role. Interpersonal theory suggests that variation in interpersonal difficulties have two main dimensions, termed agency and communion. The present study compared these dimensions of parental interpersonal problems to parental depressive symptoms as predictors of adolescent-reported parent-adolescent conflict, in a sample of 100 parents of 60 adolescents with a Major depressive disorder (92% female). We employed Bayesian multilevel modelling, leave-one-out cross-validation and model stacking to compare and weight different models. These were models predicting parent-adolescent conflict from parental depressive symptoms and from parental interpersonal problems, with and without interactions with parent gender. Results suggest that including parental interpersonal problems contributes substantially to accurate predictions of parent-adolescent conflict, and that these associations do not depend on parent gender. When parents reported more interpersonal problems related to excessive dominance or submissiveness, adolescent report of conflict tended to be higher or lower, respectively. Parental interpersonal difficulties related to the communion dimension was not associated with parent-adolescent conflict. Parental depressive symptoms were found to be negatively associated with parent-adolescent conflict in father-adolescent relationships only. These findings support the view that parental difficulties in negotiating the normative transition to a less hierarchical parent–child relationship may be related to heightened parent-adolescent conflict in adolescent depression. The study is limited by a small sample size and low number of male adolescents. Future studies on parent-adolescent conflict should consider using the interpersonal circumplex and related measures, as a theoretically rich and differentiated model of parent and adolescent interpersonal styles.

## Electronic supplementary material

Below is the link to the electronic supplementary material.

(TIF 433 kb)

(TIF 688 kb)
