# Supplementary figures and images for: Conflict with Parents in Adolescent Depression: Associations with Parental Interpersonal Problems and Depressive Symptoms
Source: Child Psychiatry Hum Dev. 2020 Jan 18;51(3):442–52. doi: 10.1007/s10578-020-00955-0 (PMC7235051; doi:10.1007/s10578-020-00955-0)

1

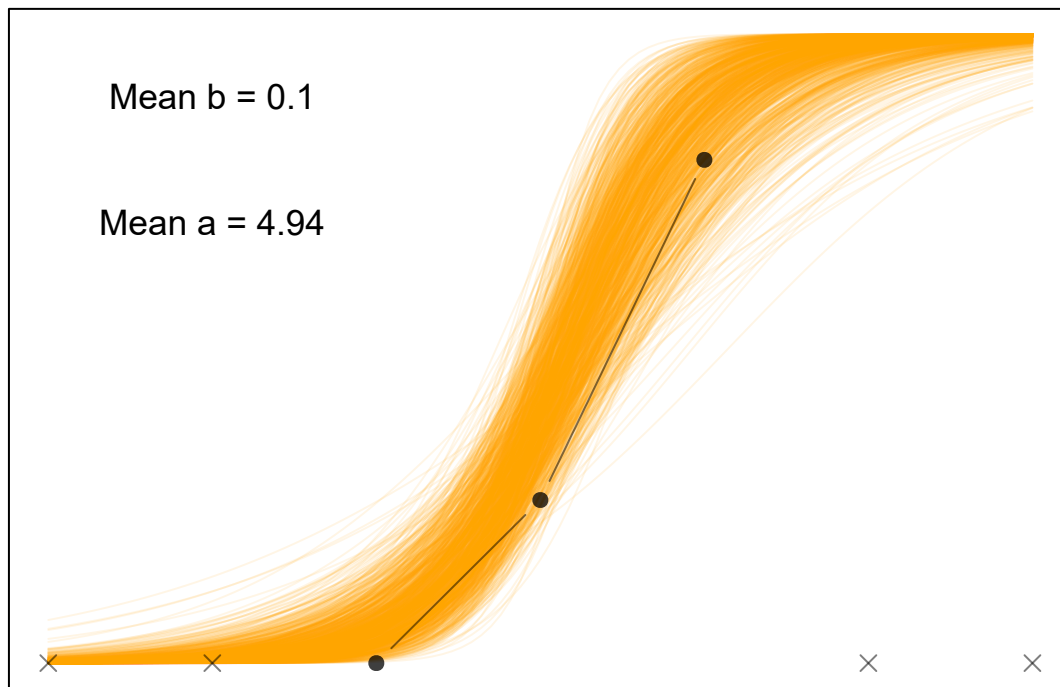

2

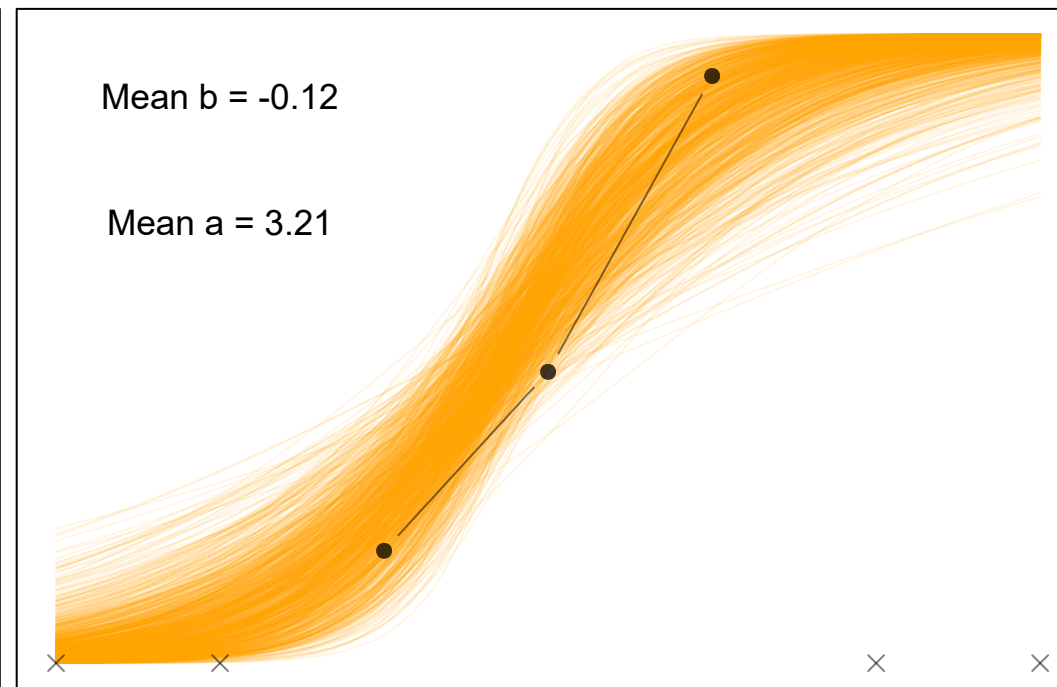

3

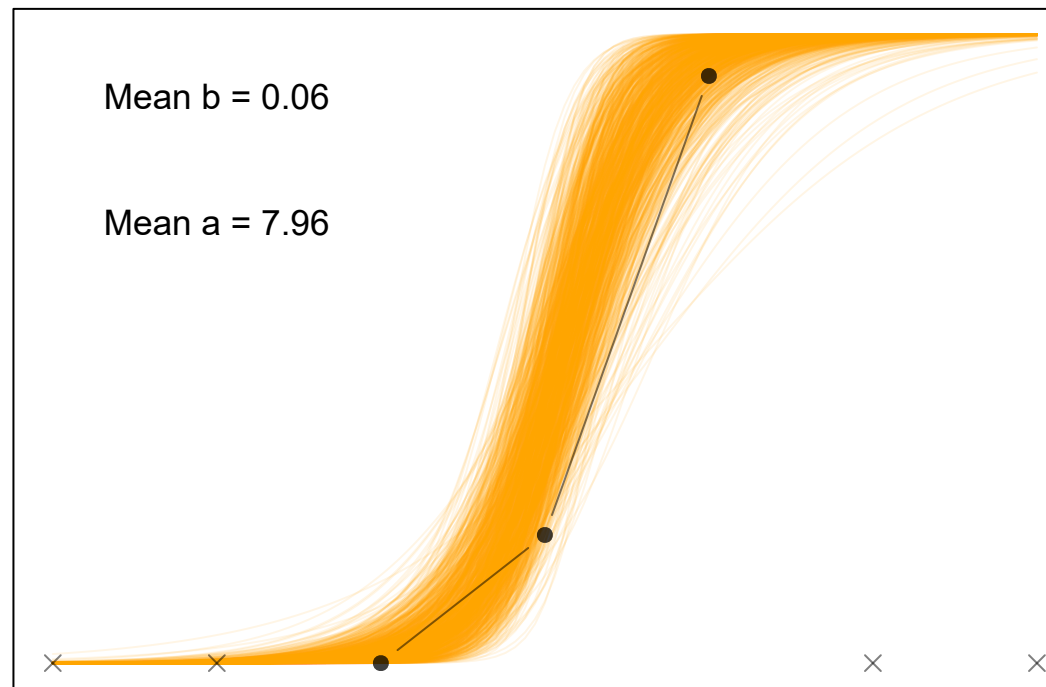

4

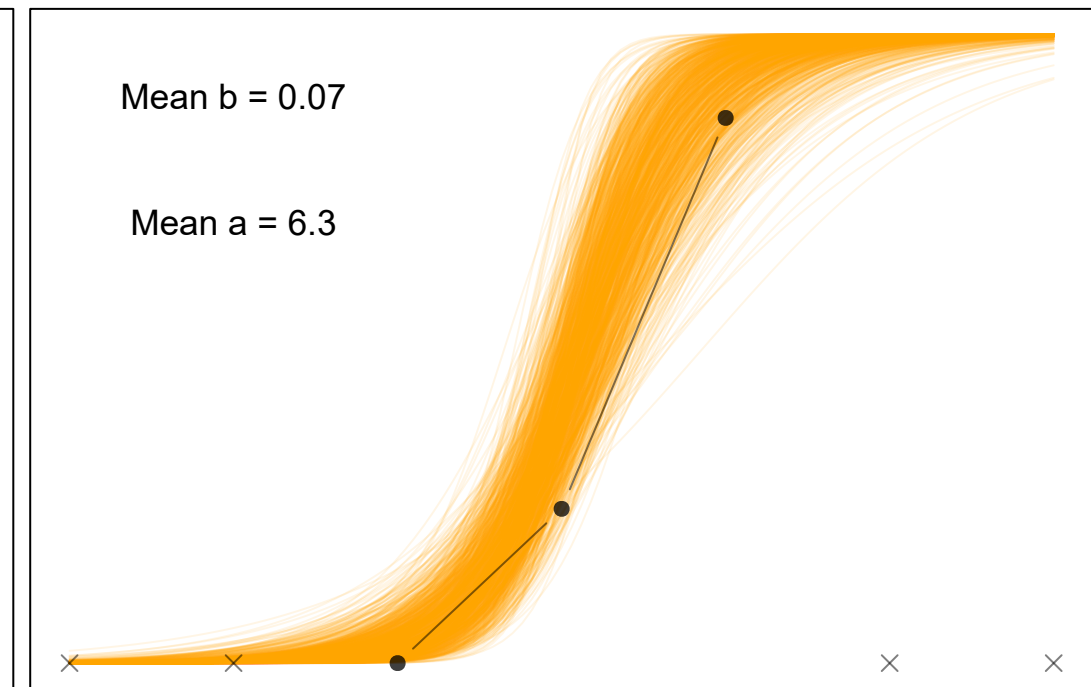

5

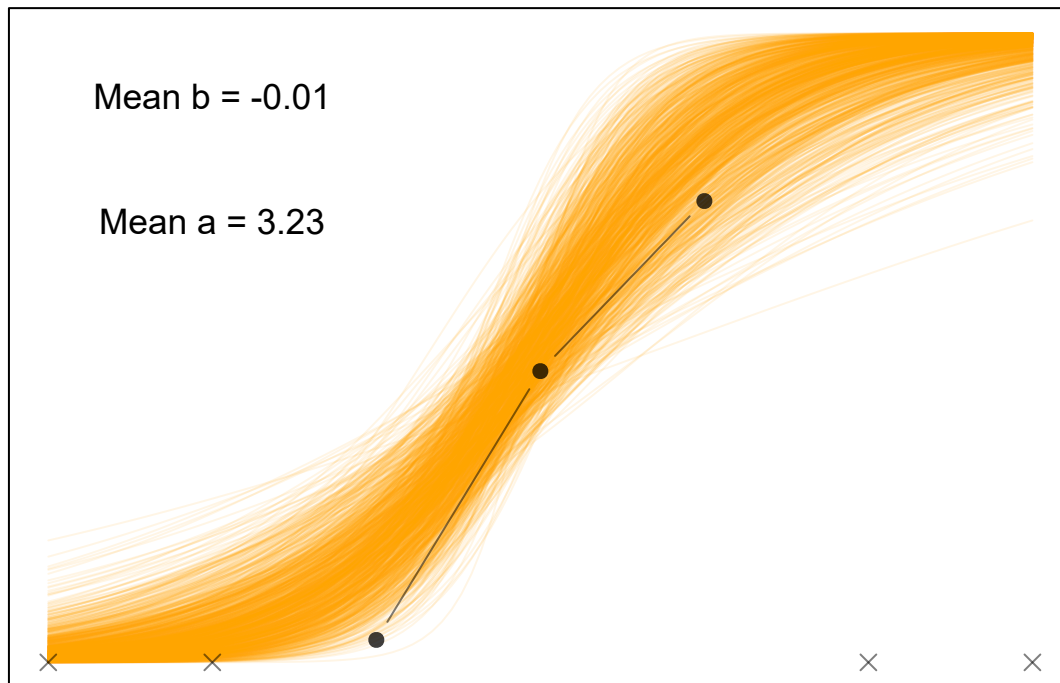

6

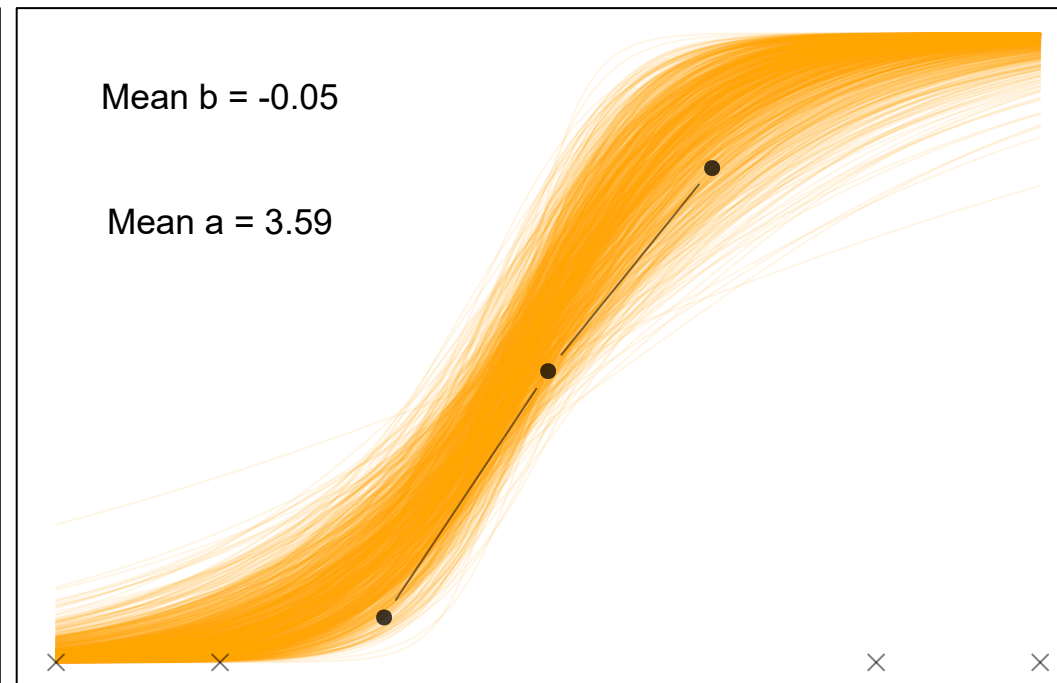

7

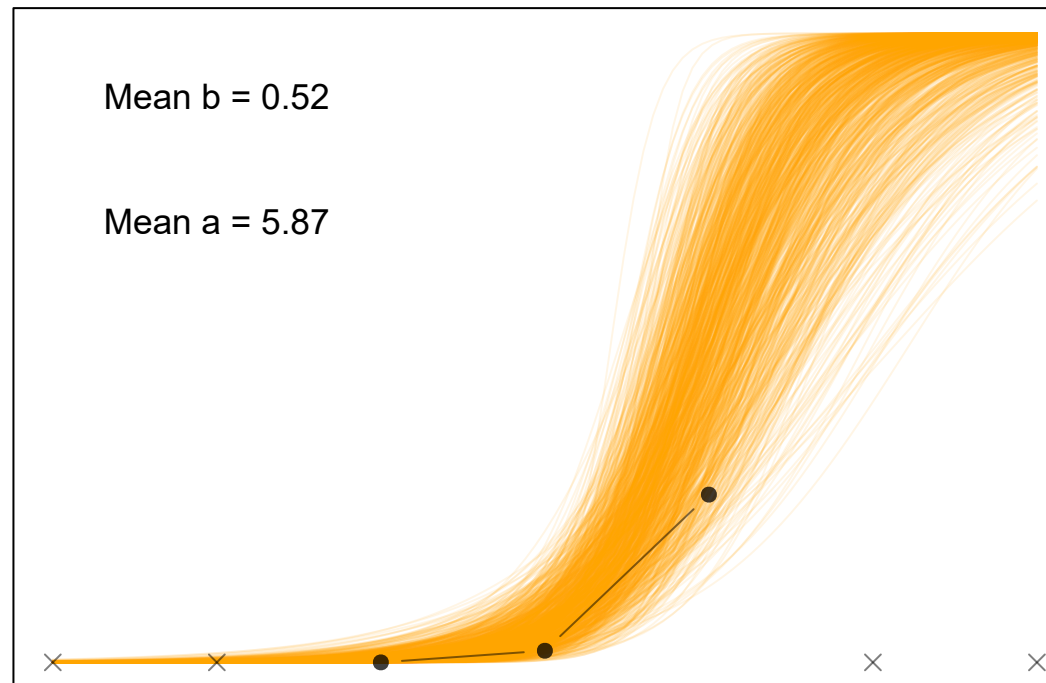

8

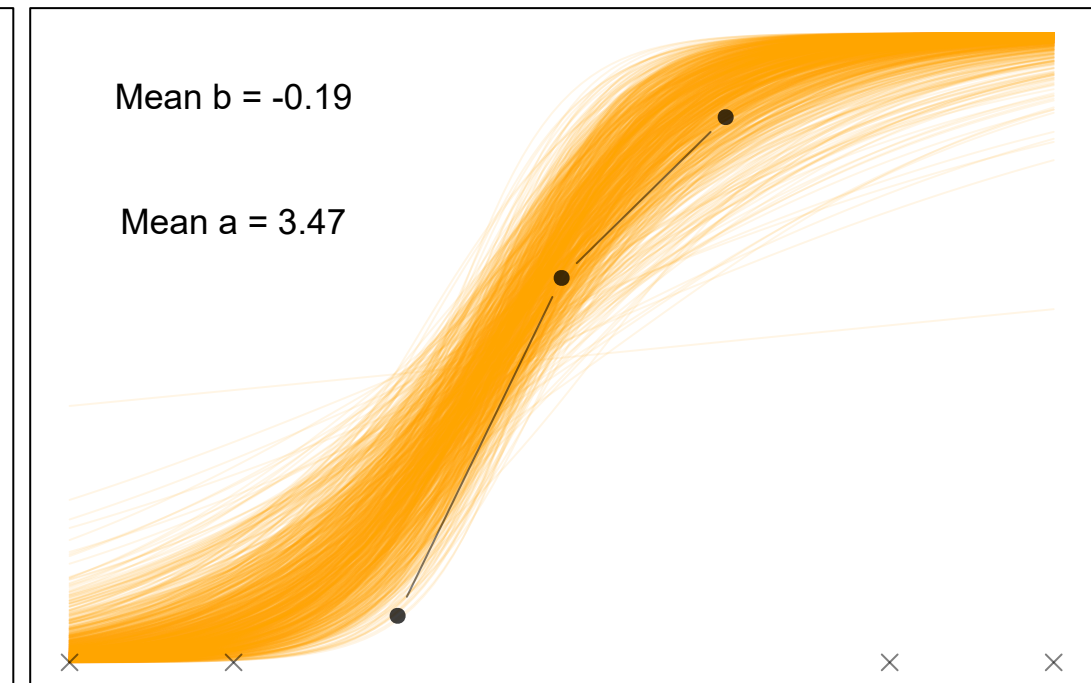

9

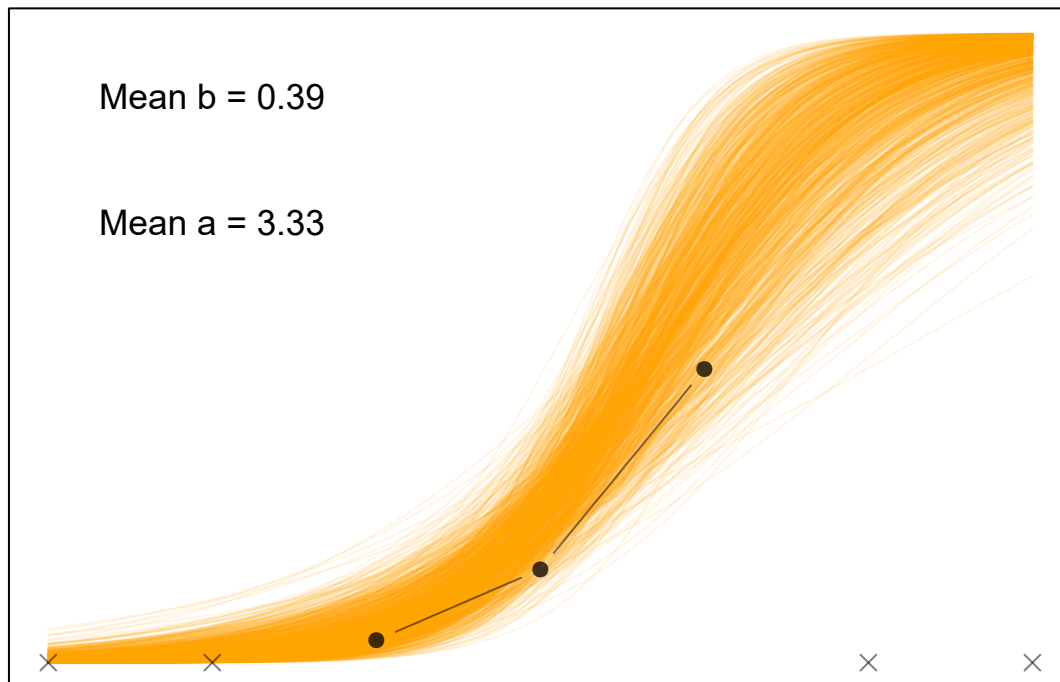

10

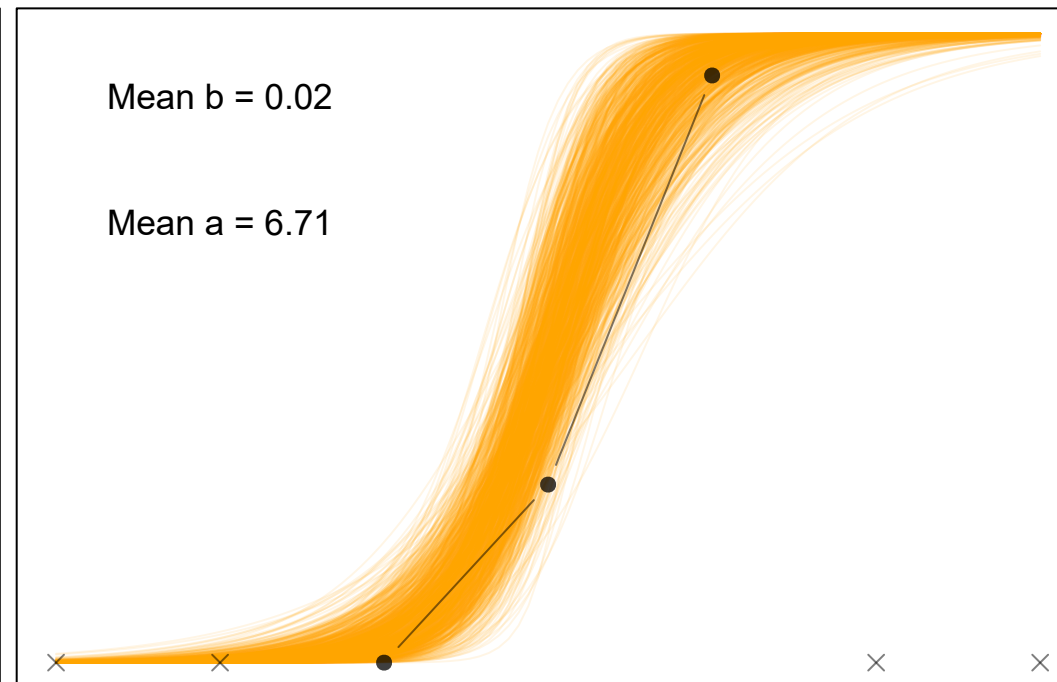

11

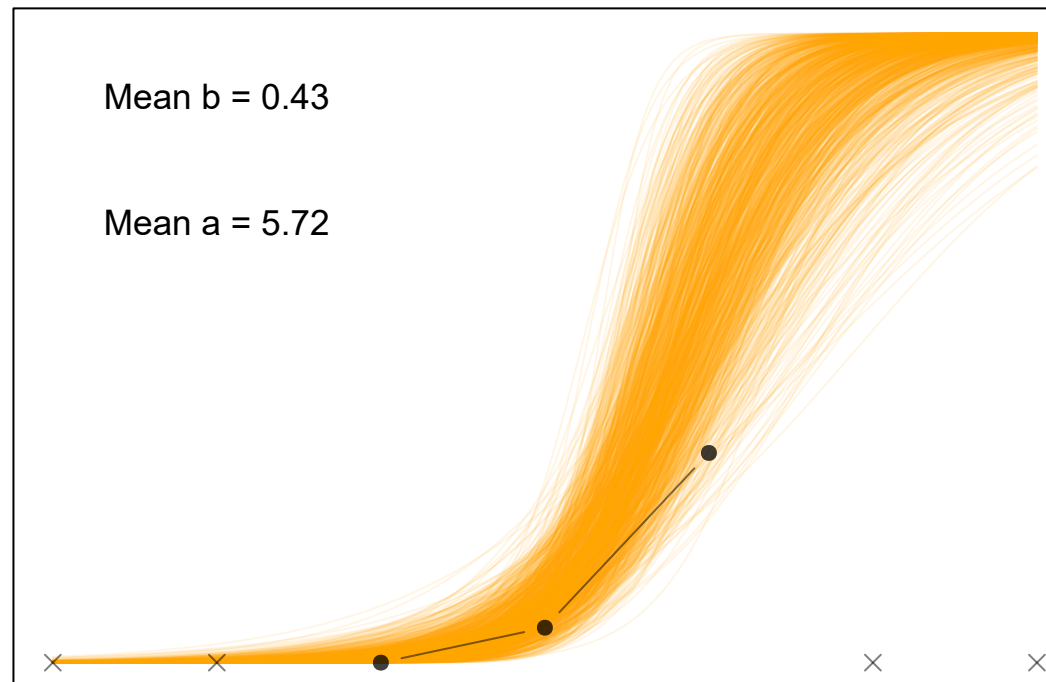

12

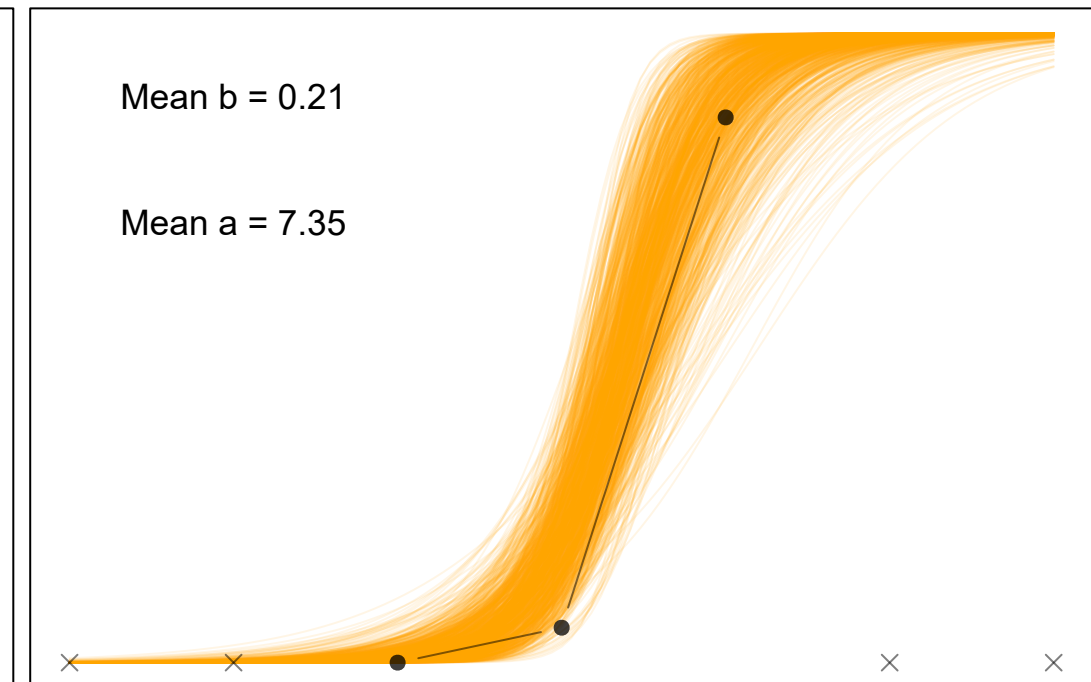

13

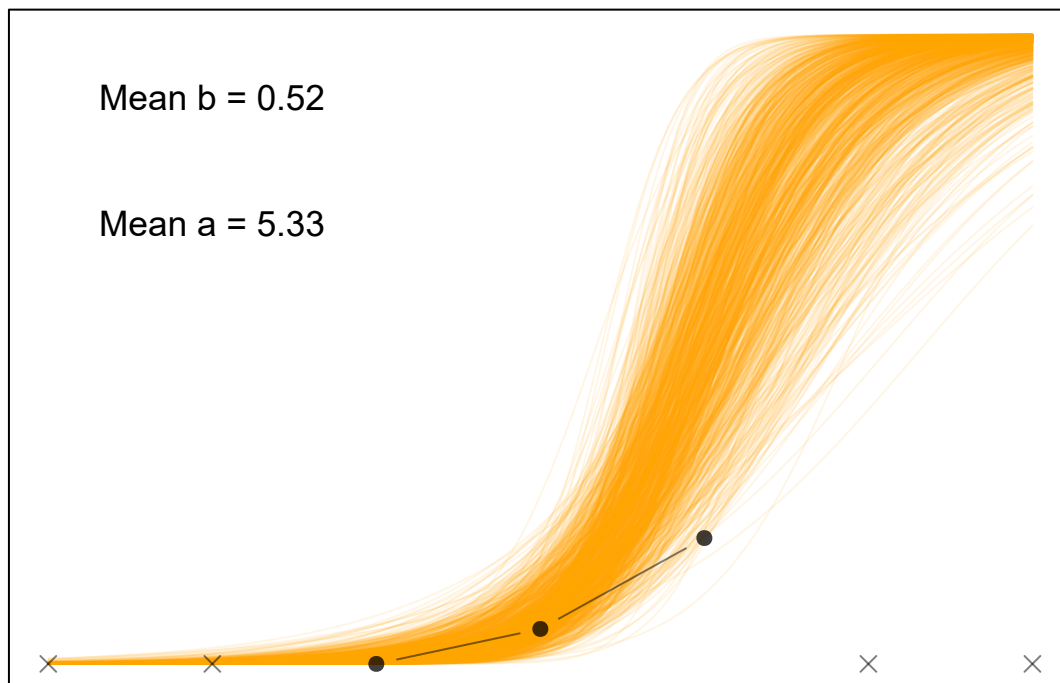

14

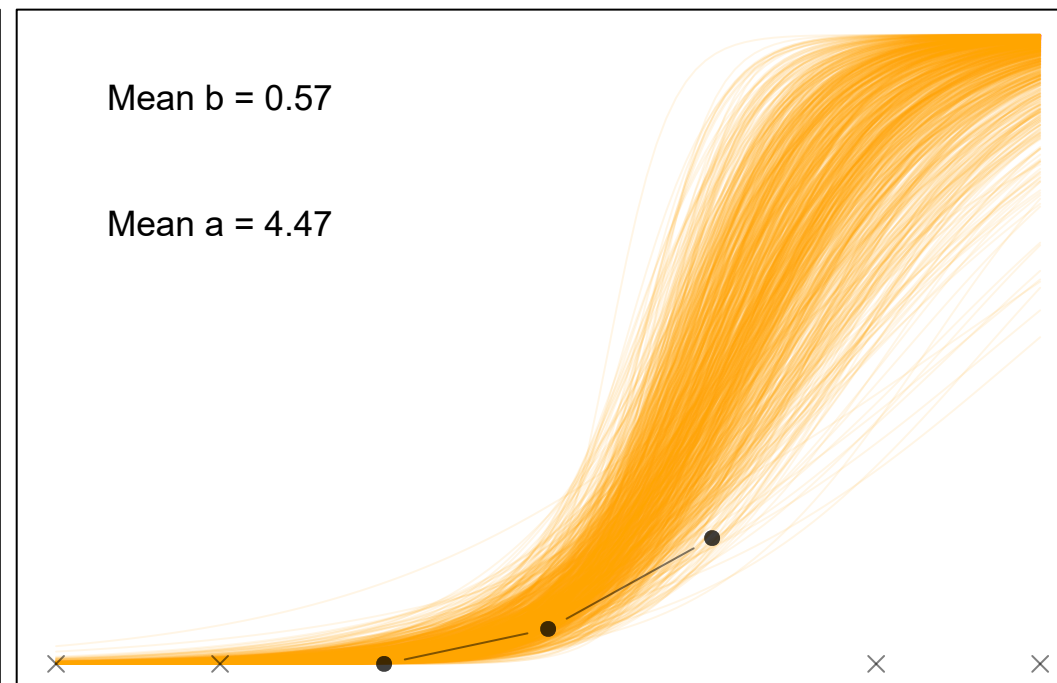

15

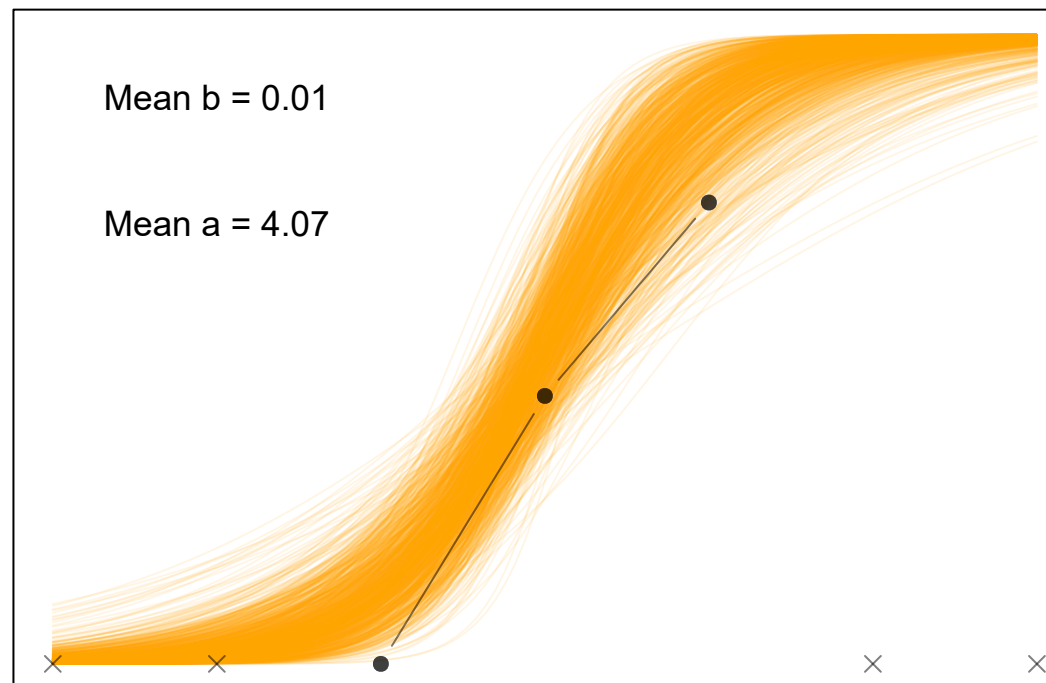

16

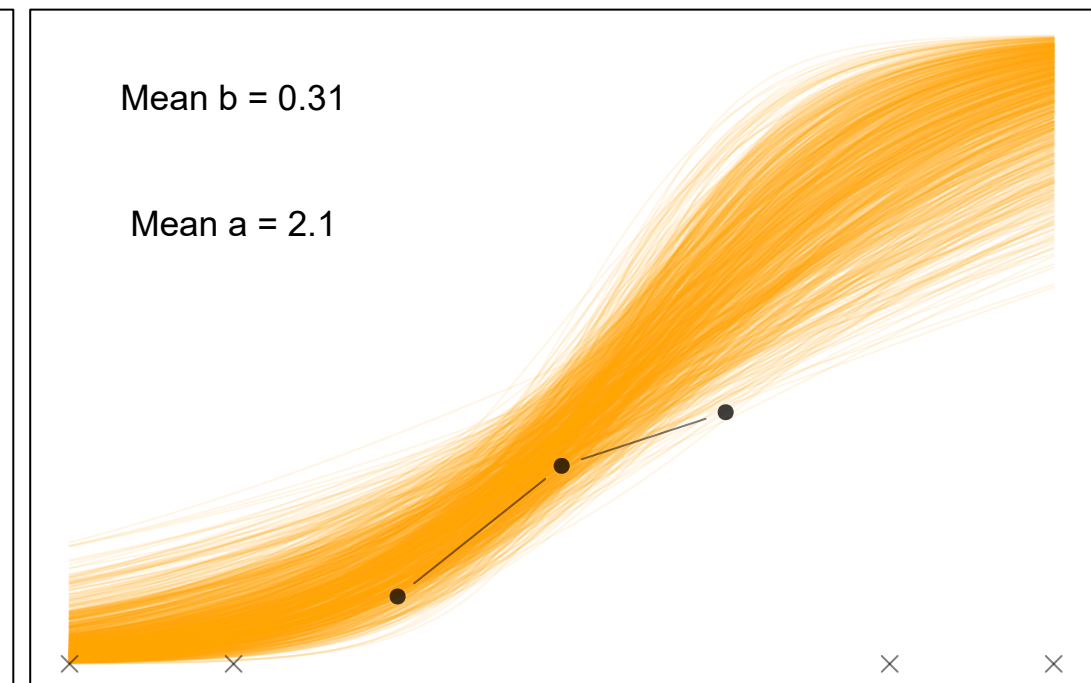

Supplement: Supplementary file 1 — (TIF 433 kb) [file 10578_2020_955_MOESM1_ESM.pdf]
